# Tri-State Medical Society of Alabama, Georgia and Tennessee

**Published:** 1892-08

**Authors:** W. E. B. Davis, F. T. Smith

**Affiliations:** Rome, Ga., President; Chattanooga, Secretary


### Society Notes.

### TRI-STATE MEDICAL SOCIETY OF ALABAMA, GEORGIA AND TENNESSEE.

We are in receipt of an abstract of the proceedings of the Tri-State Medical Society which contains an abstract of all the papers read at the last meeting and the discussion thereon. The advantage of an abstract of this kind is that it is not necessary to read through all the papers to get the leading ideas advanced by the authors. It gives us pleasure to note the rapid progress made by this young and vigorous Society.

It is wielding a powerful influence for the good of ethical medicine in our South land. Its members number many noted men of all sections.

We acknowledge the receipt also of the following reprints: "The Lacerated Cervix," "Bromide of Ethyl as an Anæsthetic," "Should not Oculists be more Careful in Prescribing Colored Glasses?" "Evolution from a Scientific Standpoint." Any of the above will be sent on application to the Secretary, Dr. Frank Tester Smith, Chattanooga, Tenn.

The next meeting will be held in Chattanooga, beginning Oct. 25th—Dr. W. E. B. Davis, Rome, Ga., President, and Dr. F. T. Smith, Chattanooga, Secretary.

---

The Wood County Medical Society, at a recent meeting, elected the following officers for ensuing year: President, Dr. Sam H. Hart; Vice-President, Dr. J. P. Wilson; Secretary, Dr. R. O. Connell; Treasurer, Dr. Cooper.

